# Are long-lasting nonspecific symptoms related to vitamin D deficiency among older adults living in nursing homes?

**DOI:** 10.1186/s12877-025-06132-z

**Published:** 2025-06-24

**Authors:** Rebeka Arnljots, Egill Snaebjörnsson Arnljots, Jörgen Thorn, Marie Elm, Michael Moore, Pär-Daniel Sundvall

**Affiliations:** 1 Research, Education, Development & Innovation, Primary Health Care, Region Västra Götaland, Sweden; 2 General Practice/Family Medicine, School of Public Health and Community Medicine, Institute of Medicine, Sahlgrenska Academy, University of Gothenburg, Gothenburg, Sweden; 3 Närhälsan Heimdal Health Care Center, Borås, Sweden; 4 Centre for Antibiotic Resistance Research (CARe), University of Gothenburg, Gothenburg, Sweden; 5 Närhälsan, Region Västra Götaland, Sweden; 6 Health Care Unit, Borås Municipality, Sweden; 7 Academic Unit of Primary Care and Population Sciences, Faculty of Medicine, University of Southampton, Aldermoor Health Centre, Aldermoor Close, Southampton, UK; 8 Närhälsan Sandared Health Care Center, Sandared, Sweden

**Keywords:** Primary care, Vitamin D, Frail older adults, Homes for the aged, Nursing homes, Angers, Anxiety, Fatigue, Confusion

## Abstract

**Background:**

Confusion, restlessness, and fatigue are common among older adults living in nursing homes. These nonspecific symptoms are often treated with antibiotics since they are frequently misinterpreted as urinary tract infections. Therefore, it is crucial to investigate other potential causes of these nonspecific symptoms. Impaired cognitive function in older adults may be associated with vitamin D deficiency and could lead to nonspecific symptoms. Currently, it is unknown whether there is a correlation between nonspecific symptoms, often misinterpreted as acute cystitis, and vitamin D deficiency.

**Methods:**

A cross-sectional study in which blood samples were collected from residents of 22 Swedish nursing homes for 25OHD (25-hydroxyvitamin D) analysis. Demographics and presence of nonspecific symptoms, such as: fatigue, restlessness, confusion, aggressiveness, reduced appetite, tendency to fall or a sense of not being oneself as well as symptom duration, were registered. Exclusion criteria: incontinence, dementia too severe to cooperate when taking a blood test, terminally ill or refusing participation. Logistic regressions were performed to determine if nonspecific symptoms persisting ≥ 3 months were associated with vitamin D deficiency.

**Results:**

Out of 901 residents in 22 nursing homes blood samples were taken from 545 participants, of whom 370 (68%) were women. The mean age was 86 years (SD 6.9), and 55% (299/545) had dementia. The prevalence of symptoms persisting ≥ 3 months was: fatigue 49% (268/545), restlessness 50% (270/545), confusion 53% (287/545), agitation/anger 47% (258/545), reduced appetite 45% (247/545), tendency to fall 48% (260/545), and a sense of not being oneself 35% (191/545). The 25OHD concentrations did not differ between various nonspecific symptoms. When adjusting for age, gender and dementia there was no association between any of the nonspecific symptoms and 25OHD.

**Conclusions:**

Nonspecific symptoms persisting ≥ 3 months and vitamin D deficiency were common among older adults living in nursing homes. However, there was no association between these long-lasting nonspecific symptoms and the concentration of vitamin D. In further research it is important to study other potential causes of nonspecific symptoms in older adults.

## Background

As the population ages, the healthcare needs of older adults have become an increasingly important focus. Older individuals often present with atypical or nonspecific symptoms when experiencing common health issues, which can complicate diagnosis and treatment [1, 2]. This is particularly true in older adults living in nursing homes, where frailty, comorbidities, and cognitive decline are prevalent. Nonspecific symptoms, such as confusion or fatigue, may obscure the underlying cause of illness, leading to potential misdiagnosis [3, 4].

Furthermore, the rising prevalence of antibiotic resistance has prompted a critical review of how antibiotics are used, particularly in the care of older adults in nursing homes [5]. As such, there is a growing need to understand and explore other potential explanations for these nonspecific symptoms in this vulnerable population.

Confusion, restlessness, and fatigue are common nonspecific symptoms among older adults living in nursing homes [1, 2] and, in the absence of new symptoms from the urinary tract, often misinterpreted as urinary tract infections (UTIs), leading to unnecessary antibiotic treatment [3, 4]. This practice increases the risk of selecting antibiotic-resistant bacteria. UTIs are more prevalent among women in nursing homes [3]. According to international consensus, even in older residents in nursing homes, symptoms from the lower urinary tract should be present to diagnose acute cystitis [6–8]. Despite this, few patients treated for suspected UTIs in nursing homes actually exhibit lower urinary tract symptoms [9, 10]. The diagnosis is instead based on nonspecific symptoms, which are not specific to the urinary tract, such as confusion, restlessness, fatigue, agitation, reduced appetite, tendency to fall, or a sense of not being oneself [11–13]. Given the growing problem of antibiotic-resistant bacteria it is important to avoid unnecessary antibiotic use [5]. Therefore, it is important to investigate alternative explanations for these nonspecific symptoms. Vitamin D deficiency is common among older adults living in nursing homes [14–16] and is associated with risk of falls [17]. It has also been linked to impaired cognitive functions and dementia [18–22]. Impaired cognitive function can manifest as nonspecific symptoms, highlighting the importance of investigating whether there is an association between these symptoms, often misinterpreted as UTIs, and vitamin D deficiency.

The aim of this study was to determine if nonspecific symptoms among older adults living in nursing homes are associated with vitamin D deficiency.

## Methods

This study was part of a larger project, with joint data collection, studying vitamin D deficiency, interleukin-6 concentrations in urine, and antimicrobial resistance in urinary pathogens among residents in Swedish nursing homes [1, 21, 23]. Throughout the initial three months of 2012, a case report form was filled out, and blood samples were obtained from all eligible residents in 22 nursing homes. These nursing homes were located in two municipalities, both rural and urban, in Region Västra Götaland, southwest Sweden, where almost all the nursing homes participated. The attending nurses were given comprehensive verbal and written instructions regarding the study procedures. The Regional Ethical Review Board of Gothenburg University approved the study (Reference number: 578 − 11).

### Inclusion and exclusion criteria

All residents permanently residing in the nursing homes for older adults, irrespective of gender or length of stay, were given the opportunity to participate. Inclusion criteria for those who accepted participation were:

- presence at a nursing home for the older adult during the study
- residents with dementia if they or their relatives did not object to participation
- residents with dementia who were able to cooperate during sampling

The criteria for exclusion were:

- residents with severe dementia that hindered collaboration during sampling
- residents in end-of-life care

### Statement of consent

All residents were briefed on the study through both verbal and written communication. For those able to make decisions and opting study participation, informed consent was obtained. However, a significant portion of participants included were individuals with varying degrees of dementia. If a resident lacked the capacity to comprehend the information and, as a result, had diminished decision-making capability, their participation was contingent upon two conditions: no objection to study participation, nor objection from appointed representatives or family members, after being informed about the study. This procedure received approval from the Regional Ethical Review Board of Gothenburg University.

### Case report form

A scheduled date was established for the collection of blood samples from each participating resident. On the same day, the attending nurse recorded information in the case report form, including age, gender, nursing home/ward, whether the resident had vitamin D supplementation, presence of a diagnosis of dementia, as well as the occurrence and duration of nonspecific symptoms: fatigue, restlessness, confusion, agitation/anger, tendency to fall, and a sense of not being oneself. The diagnosis of dementia in the medical records necessitated a comprehensive assessment, including a detailed medical history, physical examination conducted by a physician, laboratory tests, cognitive function assessment, and, if present, neuroimaging.

### Laboratory tests

As part of the larger project, the attending nurse obtained blood samples from each participating resident, and the concentrations of 25OHD in these samples were analysed at the accredited clinical chemistry laboratory of Södra Älvsborg Hospital in Borås, Sweden, using standard clinical procedures. The blood samples were cooled before transportation and typically reached the laboratory within 24 h. The measurement of 25OHD concentrations in serum was conducted using the LIAISON^®^ 25 OH Vitamin D TOTAL Assay by DiaSorin Inc., Stillwater, USA. This assay employed chemiluminescent immunoassay (CLIA) technology for the quantitative determination of 25OHD in human serum. The DiaSorin LIAISON^®^ 25 OH Vitamin D assay had a measuring range of 4.0–150 ng/mL.

### Statistical analysis

The descriptive statistics illustrated the population and proportion of residents who had experienced each nonspecific symptom for ≥ 3 months, as well as the median regarding the concentration of vitamin D in these groups. Logistic regressions were conducted to examine whether nonspecific symptoms ≥ 3 months were associated with vitamin D deficiency. For each nonspecific symptom a logistic regression analysis was performed with the symptom as the dependent variable, and the independent variables being gender, age, diagnosis of dementia, and vitamin D concentrations as a continuous variable.

A significance level of *p* < 0.05 was deemed statistically significant. Statistical analysis was performed using IBM SPSS Statistics version 29.

Power calculation was performed with level of significance 0.05, power 95%, and two-tailed hypothesis testing. It was hypothesized that 40% of residents would experience vitamin D deficiency (25OHD < 25nmol/l). A significant distinction would be that 35% of residents without any of the nonspecific symptoms would have vitamin D deficiency, whereas 55% of residents with any of the nonspecific symptoms would have vitamin D deficiency. Additionally, it was assumed that 40% of residents would exhibit any of the nonspecific symptoms. Therefore, the required sample size was 334 residents. Considering the uncertainty surrounding the assumptions, it was reasonable to include 500–600 residents in the study.

## Results

### Studied population

Out of 901 residents in 22 nursing homes, 836 met the inclusion criteria, and 596/836 (71%) chose to participate. Blood samples were collected, and study forms were completed for 545 residents, of whom 370 (68%) were women and the average age was 86 (SD 6.9). Of the 545 participants, 55% had dementia (Table 1). A detailed description of the studied population has been published previously [21].

**Table 1:** Prevalence of symptoms and 25-hydroxyvitamin D concentrations

	Prevalence of symptoms	25OHD^a^ nmol/L	Age	Female gender	Dementia
	% (n/n)	Median (IQR^b^)	Mean (SD)	% (n/n)	% (n/n)
Fatigue ≥ 3 months	49% (268/545)	27 (20–42)	86 (6.8)	68% (182/268)	44% (119/268)
No fatigue, or fatigue < 3 months	51% (277/545)	27 (20–38)	87 (7.0)	68% (188/277)	54% (150/277)
Restlessness ≥ 3 months	50% (270/545)	26 (20–37)	86 (6.7)	67% (182/270)	44% (118/270)
No restlessness, or restlessness < 3 months	50% (275/545)	28 (21–42)	86 (7.0)	68% (188/275)	53% (147/275)
Confusion ≥ 3 months	53% (287/545)	26 (20–40)	86 (6.6)	67% (193/287)	40% (114/287)
No confusion, or confusion < 3 months	47% (258/545)	28 (21–41)	86 (7.1)	69% (177/258)	49% (126/258)
Agitation/anger ≥ 3 months	47% (258/545)	26 (20–39)	86 (6.7)	67% (172/258)	43% (112/258)
No agitation/anger, or agitation/anger < 3 months	53% (287/545)	27 (21–41)	86 (7.0)	69% (198/287)	53% (153/287)
Reduced appetite ≥ 3 months	45% (247/545)	27 (20–41)	86 (6.7)	66% (163/247)	44% (108/247)
No reduced appetite, or reduced appetite < 3 months	55% (298/545)	27 (21–40)	86 (7.0)	69% (207/298)	54% (160/298)
Tendency to fall ≥ 3 months	48% (260/545)	26 (20–42)	86 (6.5)	69% (178/260)	42% (110/260)
No tendency to fall, or tendency to fall < 3 months	52% (285/545)	28 (21–40)	86 (7.2)	67% (192/285)	52% (149/285)
Sense of not being oneself ≥ 3 months	35% (191/545)	26 (20–35)	86 (7.0)	69% (132/191)	40% (77/191)
No sense of not being oneself, or sense of not being oneself < 3 months	65% (354/545)	28 (21–41)	86 (6.8)	67% (238/354)	52% (185/354)

^a^ 25-hydroxyvitamin D

^b^ Interquartile range

### Prevalence of symptoms

The prevalence of symptoms persisting ≥ 3 months among the 545 residents were: fatigue 49%, restlessness 50%, confusion 53%, agitation/anger 47%, reduced appetite 45%, tendency to fall 48%, and a sense of not being oneself 35% (Table 1).

### Serum 25OHD concentrations

The mean concentration of 25OHD in serum for all participants was 34 nmol/L (SD 21, median 27, range 4–125), as previously published [21]. The 25OHD concentrations did not differ between various nonspecific symptoms (Table 1).

### Factors associated with nonspecific symptoms persisting ≥ 3 months

There was no association between any of the nonspecific symptoms persisting ≥ 3 months and vitamin D concentrations when adjusting for age, gender, and presence of dementia (Table 2).

**Table 2:** Factors associated with nonspecific symptoms lasting ≥ 3 months

	Adjusted odds ratio (aOR) with 95% confidence interval for association with nonspecific symptoms^a^			
	25OHD^b^ (nmol/L) aOR (95% CI), p-value	Increasing age in years aOR (95% CI), p-value	Male gender aOR (95% CI), p-value	Dementia aOR (95% CI), p-value
Fatigue ≥ 3 months	1.0 (0.99–1.0), *p* = 0.87	0.98 (0.96–1.0), *p *= 0.18	0.97 (0.67–1.4), *p *= 0.88	1.0 (0.73–1.5), *p *= 0.86
Restlessness ≥ 3 months	1.0 (0.99–1.0), *p* = 0.28	1.0 (0.97–1.0), *p *= 0.76	1.0 (0.71–1.5), *p* = 0.89	1.1 (0.76–1.5), *p* = 0.67
Confusion ≥ 3 months	1.0 (0.99–1.0), *p *= 0.89	0.99 (0.97–1.0),* p* = 0.55	1.1 (0.75–1.6), *p* = 0.68	1.6 (1.1–2.2), *p* = 0.011
Agitation/anger ≥ 3 months	1.0 (0.99–1.0), *p* = 0.49	1.0 (0.98–1.0), *p *= 0.90	1.1 (0.77–1.6),* p *= 0.56	1.1 (0.80–1.6), *p *= 0.50
Reduced appetite ≥ 3 months	1.0 (0.99–1.0), *p* = 0.88	1.0 (0.97–1.0), *p* = 0.78	1.2 (0.82–1.7), *p* = 0.39	1.1 (0.79–1.6), *p* = 0.53
Tendency to fall ≥ 3 months	1.0 (0.99–1.0), *p *= 0.74	1.0 (0.98–1.0), *p* = 0.69	0.97 (0.67–1.4), *p* = 0.87	1.2 (0.88–1.8), *p* = 0.22
Sense of not being oneself ≥ 3 months	0.99 (0.99–1.0), *p* = 0.18	1.0 (0.98–1.0), *p* = 0.96	0.92 (0.62–1.3), *p* = 0.65	1.3 (0.90–1.9), *p* = 0.17

^a^ Multivariable logistic regression with 545 included in the analysis

^b^ 25-hydroxyvitamin D

## Discussion

Among older adults living in nursing homes, vitamin D deficiency and nonspecific symptoms persisting ≥ 3 months were common. However, there was no association between these long-lasting nonspecific symptoms and vitamin D deficiency.

The intensity of nonspecific symptoms typically varies over time, and during episodes of increased severity nurses often misinterpret these symptoms as acute cystitis. Additionally, since 25OHD concentrations fluctuate slowly over time and potential deficiencies develop gradually, there is greater likelihood of them being associated with symptoms persisting for an extended period. Based on this reasoning, and according to the pre-specified statistical analysis plan, we analyzed long-lasting nonspecific symptoms persisting ≥ 3 months instead of symptoms of shorter duration. Exploratively, we also carried out the analysis with a cut-off for nonspecific symptoms lasting ≥ 1 month. Also with this duration cut-off there was no association between nonspecific symptoms and vitamin D concentrations.

The median concentration of vitamin D and interquartile ranges were similar across groups (Table 1). In the regression analysis (Table 2), vitamin D was treated as a continuous variable. This approach allows to maximize the use of all available data points, while adjusting for confounders like dementia (Table 2). An alternative method of categorizing vitamin D levels into predefined cut-off values would result in a loss of data granularity. For this reason, vitamin D was treated as a continuous variable. Given that none of the *p*-values in Table 2 were close to statistical significance and that the median values between groups were similar (Table 1), it is unlikely that dividing the data into categorical cut-offs would yield a different result. Furthermore, exploratory analyses using different cut-off levels would generate numerous *p*-values, raising the risk of type I errors due to multiple testing.

Due to the data being 10 years old the prevalence of vitamin D deficiency today may differ. However, the aim of this sub-study was not to describe the prevalence of vitamin D deficiency, but rather to investigate any association between low concentrations of vitamin D and presence of symptoms. Therefore, the conclusions are not influenced by potential changes in vitamin D supplementation over the years or the current prevalence of vitamin D deficiency. Furthermore, there is no recommendation for general supplementation with vitamin D of all residents in nursing homes in Sweden.

### Strengths and limitations

**Fig. 1:**
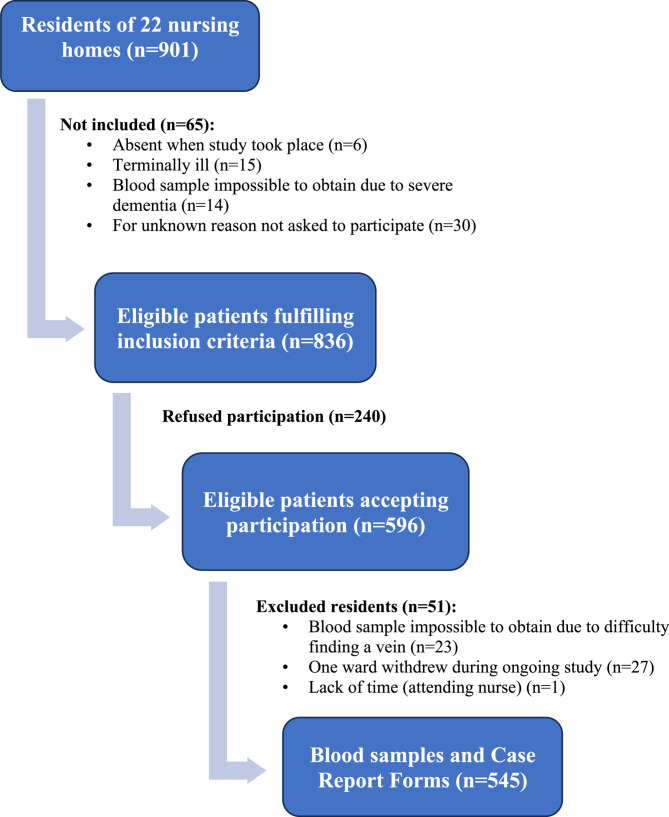
Participation flowchart

One strength of this study is that blood samples were collected from all participating residents for whom it was possible to obtain a blood sample during the study period. Case report forms and blood samples were collected from 60% (545/901) of all registered residents in the nursing homes (Fig. 1). While 60% participation may seem low it is consistent with previous studies conducted in nursing homes [14]. It is challenging to include frail older adult research participants in nursing homes and collect blood samples. A large proportion have dementia, and when the research participants cannot provide informed consent, according to ethical regulations, relatives must be contacted, informed, and asked if they do not oppose participation by their relative in the study.

Another strength is that we adjusted for dementia, in which nonspecific symptoms are common. There may be differences between the groups with and without dementia. Therefore, we used dementia as an independent variable in the regression analysis. As expected, confusion was more common in individuals with dementia, with an adjusted odds ratio of 1.6 (95% CI 1.1–1.2), *p* = 0.011 (Table 2).

Vitamin D deficiency is also more common among older adults living in nursing homes suffering from dementia, compared to older adults without dementia [18–21]. There were more women than men in this study which reflects the gender distribution among older adults living in nursing homes. In the logistic regressions we also adjusted for gender. Despite adjusting for confounders there is potential for residual confounding.

A weakness may be that nurses themselves had to determine whether residents had any of the nonspecific symptoms, as validated instruments were not used for this purpose. However, this is also a strength because the symptoms registered by nurses in this study were evaluated in the same way as by nurses in clinical practice. With or without dementia, individuals with nonspecific symptoms, such as confusion, may be unable to articulate their symptoms. Therefore, these individuals were assessed by nurses who had a thorough and comprehensive understanding of the resident’s condition.

### Clinical relevance

In everyday clinical practice it is important to note that low concentrations of vitamin D and long-lasting nonspecific symptoms are very common among older adults living in nursing homes. Efforts should be made to identify the possible cause of these symptoms and not hastily conclude that it is an acute cystitis when simultaneous lower urinary tract symptoms are absent, leading to unnecessary antibiotic treatment. Given the growing problem of antibiotic-resistant bacteria, it is crucial not to overuse antibiotics [5].

Numerous potential associations with vitamin D are described in the literature and prior research, varying in their level of substantiation [14, 17–22, 24–30]. However, it is equally important to publish articles that demonstrate conditions and factors not related to vitamin D deficiency. Thus, even if there was no association between vitamin D concentrations and nonspecific symptoms in this study, the information still contributes to the ongoing research on vitamin D.

In future studies, it is important to continue searching for other causes of these nonspecific symptoms among older adults living in nursing homes. In consideration of a population where the average age is constantly rising and life expectancy increasing, it is important to study factors that affect the health of older adults.

## Conclusions

Nonspecific symptoms persisting ≥ 3 months and vitamin D deficiency were common among older adults living in nursing homes. However, there was no association between these long-lasting nonspecific symptoms and vitamin D concentrations. Although there was no association, valuable information is still contributed to the ongoing research on vitamin D and older adults.

## Data Availability

The data supporting the conclusions of this study can be obtained from the corresponding author upon reasonable request.
